# Modeling sharp wave - ripple complexes and their interactions with cortical slow oscillations through a cortico-CA3-CA1 model

**DOI:** 10.1186/1471-2202-12-S1-O20

**Published:** 2011-07-18

**Authors:** Jiannis Taxidis, Stephen Coombes, Robert Mason, Markus Owen

**Affiliations:** 1 School of Mathematical Sciences, University of Nottingham, Nottingham, NG7 2RD, UK; 2 School of Biomedical Sciences, University of Nottingham, Medical School, Nottingham NG7 2UH, UK

## 

The hippocampus, and particularly the CA3 and CA1 areas, exhibit a variety of oscillatory rhythms that span frequencies from the slow theta range (4-10 Hz) up to fast ripples (~200 Hz). Various computational models of different complexities have been developed in an effort to simulate such population oscillations. Nevertheless the mechanism that underlies the so called sharp wave-ripple complex, observed in extracellular recordings in CA1, still remains elusive.

We present a combination of two computational models of the rat CA3 and CA1 hippocampal areas respectively. Both models are simple one-dimensional arrays of pyramidal and interneuronal populations, interacting only via fast AMPA and GABA_A_ synapses. Connectivity schemes and postsynaptic potentials are based on biological data, rendering the network topology as realistic as possible.

Both models are first validated individually by showing that they reproduce a number of established anatomical and/or functional characteristic properties of the corresponding areas, including population bursts in CA3 and gamma oscillations in CA1.

The two networks are coupled in a full CA3-CA1 model through a feedforward scheme mimicking Schaffer collaterals. Quasi-synchronous population bursts in CA3 result in transient responses in CA1, consisting of deep depolarizations in the dendritic layer accompanied by transient ~150-200 Hz field oscillations in the somatic layer. These responses are shown to accurately reproduce a number of basic characteristic features of sharp wave-ripple complexes, reported previously in a variety of neurophysiological studies. By examining these features through our model, we are led to the formulation of a novel mechanism for the generation of ripples, based purely on chemical interactions and avoiding any use of gap junctions. The depolarizing input from CA3, produces intense interneuronal firing in CA1 which is regulated and synchronized by strong, fast-decaying, recurrent inhibition, resulting in a field oscillation within the ripple frequency range. Pyramidal cells, trapped within this inhibitory barrage, have a more passive role, with a small subset of them producing most of the excitatory spikes in a ripple. Through our proposed mechanism, we are able to interpret neurophysiological observations, such as the ripple disruption by halothane and the selective firing of pyramidal cells during ripples, which has various implications on memory consolidation.

Finally, the CA3-CA1 model is coupled with a cortical network exhibiting slow oscillations, in a feedback loop involving the mossy fiber and temporoammonic pathway inputs to the hippocampus and monosynaptic projections from CA1 to prefrontal cortex. We study the resulting correlations between hippocampal and cortical activity in an effort to uncover important parameters and mechanisms on which they depend. We show how the spiking activities of CA3 and CA1 depend on the inhibition-excitation ratio, induced by the two hippocampal inputs, and how this ratio can affect established correlations between cortical UP states and ripples. Such correlations have been suggested to be important for the transferring of memories to the cortex for long-term storage.

